# Is foliar spectrum predictive of belowground bacterial diversity? A case study in a peach orchard

**DOI:** 10.3389/fmicb.2023.1129042

**Published:** 2023-02-24

**Authors:** Na Sun, Weiwei Zhang, Shangqiang Liao, Hong Li

**Affiliations:** ^1^Institute of Plant Nutrition, Resources and Environment, Beijing Academy of Agriculture and Forestry Sciences, Beijing, China; ^2^Institute of Grassland, Flowers and Ecology, Beijing Academy of Agriculture and Forestry Sciences, Beijing, China

**Keywords:** foliar spectral traits, plant productivity, fruit quality, bacterial diversity, soil conditioner

## Abstract

Rhizosphere bacteria can have wide-ranging effects on their host plants, influencing plant biochemical and structural characteristics, and overall productivity. The implications of plant-microbe interactions provides an opportunity to interfere agriculture ecosystem with exogenous regulation of soil microbial community. Therefore, how to efficiently predict soil bacterial community at low cost is becoming a practical demand. Here, we hypothesize that foliar spectral traits can predict the diversity of bacterial community in orchard ecosystem. We tested this hypothesis by studying the ecological linkages between foliar spectral traits and soil bacterial community in a peach orchard in Yanqing, Beijing in 2020. Foliar spectral indexes were strongly correlated with alpha bacterial diversity and abundant genera that can promote soil nutrient conversion and utilization, such as *Blastococcus*, *Solirubrobacter,* and *Sphingomonas* at fruit mature stage. Certain unidentified or relative abundance <1% genera were also associated with foliar spectral traits. We selected specific indicators (photochemical reflectance index, normalized difference vegetable index, greenness index, and optimized soil-adjusted vegetation index) of foliar spectral indexes, alpha and beta diversities of bacterial community, and quantified the relations between foliar spectral traits and belowground bacterial community *via* SEM. The results of this study indicated that foliar spectral traits could powerfully predict belowground bacterial diversity. Characterizing plant attributes with easy-accessed foliar spectral indexes provides a new thinking in untangling the complex plant-microbe relationship, which could better cope with the decreased functional attributes (physiological, ecological, and productive traits) in orchard ecosystem.

## 1. Introduction

### 1.1. The interaction between plant and soil microbial community

Plants, microorganisms, and soils interact with each other and unite biotic and abiotic factors into a living complexity *via* material cycling and energy flow in the terrestrial ecosystem (Wardle et al., 2004; Bardgett et al., 2006; Bardgett and Van der Putten, 2014). The interactions between plants and soil biota exert influences on ecosystem functioning and plant community (Fierer et al., 2009; Sul et al., 2013; Tedersoo et al., 2014; Kaiser et al., 2016), in undescribed and diverse ways that were poorly understood to date (Sul et al., 2013). This has induced great enthusiasm of researchers into interactions between aboveground plant productivity and belowground ecological functioning (Bardgett and Van der Putten, 2014; Leff et al., 2018; Cavender-Bares et al., 2022). Individual plant species can greatly impact soil microbial communities (Bardgett et al., 1999; Berg, 2009). Growing evidence showed discrepancies in soil bacterial and fungal communities that were linked to plant community composition at landscape (Grayston et al., 2001; de Vries et al., 2012) and global scales (Prober et al., 2015) in recent decades. Moreover, strong correlations between individual plant species and soil fungal (Lekberg and Waller, 2006), bacterial (Berg, 2009), and nematode (Bezemer et al., 2010) communities were observed in previous studies. However, it remains unclear that whether these correlations were driven by shared environmental preferences or by the direct influence from locally dominant plant species. Furthermore, plants affect soil microbial community in numerous ways, and it is not clear whether and what plant attributes could be used to predict soil microbial community (Leff et al., 2018; Cavender-Bares et al., 2022).

### 1.2. Plant traits characterize soil microbial community

The difficulty in understanding what controls co-distribution of plants and soil microbes is that plants exert influences on while subjecting to impacts from soil microbial community. The complex interactions pose an obstacle in understanding the co-distribution of plants and soil microbes. For instance, plants were reported associated with a nonrandom subset of soil microbes within distinct ecosystems (Gottel et al., 2011; Lundberg et al., 2012; Sul et al., 2013; Shi et al., 2015), while soil microbes can be differentiated within co-occurring plant species (Ishida et al., 2007; Bonito et al., 2014; Burns et al., 2015) or even different individuals within the same species (Massenssini et al., 2015; Gehring et al., 2016; Wagner et al., 2016). It has been observed that soil biota was more responsive to key functional traits of plants than to plant diversity. Foliar traits, such as biochemical and structural characteristics (Violle et al., 2007), were indicative for plant strategies (Wright et al., 2004), plant responses to environmental pressures (Garnier et al., 2007), and ecosystem processes and services (Díaz and Cabido, 2001; Lavorel and Garnier, 2002; Lavorel et al., 2011). Therefore, ecosystem management and studies are increasingly using foliar traits (Kokaly et al., 2003; Douma et al., 2012). However, the conditional acquisition of essential foliar traits is costly, time-consuming, and notoriously difficult to acquire, especially in remote areas (Schweiger et al., 2017).

### 1.3. Foliar spectral traits and their applications

The distinct spectral responses of vegetation are determined by biochemical and structural traits of plant tissues, and the three-dimensional structure of the canopy, which could be predicted by imaging spectroscopy that captures the spectral response in narrow, spectrally contiguous bands (Schaepman, 2007; Sul et al., 2013; Asner et al., 2017). Certain spectral bands were proved sensitive to biochemical and structural traits of plant tissues (Curran, 1989). For instance, foliar trait could influence its spectral properties: reflectance, transmittance, and absorbance of light (Ustin et al., 2009; Sul et al., 2013; Schneider et al., 2017; Schweiger et al., 2018). Hence, by measuring foliar spectral properties using, for example, spectroscopy (many adjacent spectral bands with high spectral resolution), foliar traits may be approximated easily and low-cost (Sul et al., 2013). Foliar spectral properties determined by a field spectrometer or airborne remote sensing have been widely used for individual sunlit top of canopy leaves in forest ecosystem (Asner et al., 2011; Doughty et al., 2017), and aboveground biomass of prairie ecosystems (Cavender-Bares et al., 2021). Little is known about the spectral properties in orchard ecosystem (Liu, 2022), which harbor a diverse microbial community that can be affected by tillage, microbicide application, soil fumigation, and fertilization (Peck et al., 2011; Sul et al., 2013; Wang et al., 2017; Liang et al., 2018). Monitoring plant (community) attributes has dramatically improved in recent decades with advances in fine-grain mapping of canopy structure and chemistry (Cavender-Bares et al., 2022), which could benefit the investigation of plant-microbe relationship in the orchard ecosystem.

Considering the feedback process between orchard ecosystem productivity and belowground bacterial composition and diversity, we hypothesized that plant productivity of orchard ecosystem would be detectable with field spectrometer and could be used to predict belowground bacterial diversity. To test this hypothesis, we adopted root-zone ecological restoration practices (RERP) with soil conditioner or organic fertilizer at the end of growing season to realize differentiated soil bacterial communities in a peach orchard in Beijing. Our previous results demonstrated that RERP increased bacterial diversity and altered bacterial taxonomy and metabolic functions *via* improved soil properties (balanced nutrients, decreased bulk density, and increased water holding capacity) at soil depth of 20–40 cm (Sun et al., 2022). In this study, soil bacterial diversity (0–20 cm and 20–40 cm), plant productivity, and the parallel foliar spectral profiles were obtained at fruit mature stage of the following growing season after treatment, with objectives as follows: (1) clarify which foliar spectral indexes could represent peach productivity; (2) test whether aboveground plant productivity, as detected from foliar spectral indexes, could predict soil bacterial processes and community composition; and (3) determine to what extent of belowground soil bacterial alpha and beta diversity processes could be inferred by foliar spectral indexes. Quantified linkage between foliar spectral indexes with soil bacterial diversity attributes could provide an easy access to belowground bacterial community for better coping with the decreased functional attributes (physiological, ecological, and productive traits) in degraded orchard ecosystem.

## 2. Materials and methods

The experimental peach orchard (20 ha) was established in 2008 on meadow cinnamon soil in Yanqing District, Beijing (115.99E, 40.53 N), with a warm, temperate, continental monsoon climate. The experimental location has an average annual temperature of 8.5°C and an average annual precipitation of 443 mm. Planted on a flat terrain, the test peach trees all received the same environmental conditions, such as sunlight and precipitation. An early-maturing cultivar named Chunxue has been planted since 2012 in the orchard, with a row spacing of 4 m and a line spacing of 3 m. See pre-treatment soil properties of the orchard in 2020 in Supplementary Table S1.

### 2.1. Experimental treatments

A self-invented soil restoration practice-RERP (patent application No. 2021115124384) was employed in the experimental peach orchard (Sun et al., 2022). RERP can increase the productivity of the peach orchard (reflecting in fruit yield and quality) by improving soil physical, chemical, and microbial properties in the root zone. In order to implement RERP, a trench (12 m long, 0.8 m wide, and 0.6 m deep) that was 1.2 m apart from the west side of the trees were dug out in October 2020. The dug-out soil was placed in three piles and filled back to the trench according to soil depths (0–20 cm, 20–40 cm, and 40–60 cm). There were three treatments that applied on a total of 45 trees, with three replications for each treatment. Border rows were arranged around test trees. Treatment 1 (T1) was RERP with soil conditioner (3 t ha^−1^), organic fertilizer (15 t ha^−1^ DW), and mineral fertilizer (N:P_2_O_5_:K_2_O = 15:5:10, 900 kg ha^−1^) and evenly applied at soil depths of 20, 40, and 60 cm in the trench. Treatment 2 (T2) was RERP with organic fertilizer. Application materials and methods of T2 was the same as T1, only without the application of 3 t ha^−1^ soil conditioner. Conventional practice (CK) in the orchard was considered as control, receiving 650 kg ha^−1^ of urea (46%), 600 kg ha^−1^ of calcium superphosphate, and 310 kg ha^−1^ potassium sulfate each year (applied on May and November). Pest control and regular management were conducted as required, following the local practices for all treatments.

### 2.2. Foliar spectral traits and fruit attributes determination

Foliar spectral traits were measured at mature fruit stage (July 19, 2021) on a sunny day during 10:00–11:00 am, with no irrigation or rainfall 1 week earlier. Normalized difference vegetable index (NDVI), greenness index (GI), optimized soil-adjusted vegetation index (OSAVI), photochemical reflectance index (PRI), normalized pigment chlorophyll index (NPCI), modified chlorophyll absorption ratio index (MCARI), and structure insensitive pigment index (SIPI) were measured on the 7^th^ or 8^th^ functional leaves on both south and north sides of each tree, using a potable plant reflectance spectrometer (PolyPen RP-410, Photon System Instruments, Drasov, CZ). Foliar spectral indexes were averaged over six reads from three trees (in one block) for each replication. Equations for calculation of these indexes are listed in Supplementary Table S2. Computed data of these indexes were downloaded from the reflectance spectrometer *via* SpectraPen software 1.1.0.14 (Photon System Instruments, Drasov, CZ).

For each treatment, a total of 18 mature fruits were taken on south and north sides of three trees for the determination of fruit attributes on 19 July 2021. Fruit attributes of each replication were determined and averaged over six fruits from three trees in one block. Fruit thinning was evenly performed on all test trees, with the same level of fruit numbers on each tree. Thereby, it is assumed that single fruit weight could represent yield. Fruit transverse diameter and vertical diameter were determined by a Vernier caliper. Total soluble solid content was determined using a refractometer (RHBO-90, Link Co. Ltd., Taiwan, China). Soluble sugar content was determined with anthrone-sulfuric colorimetric method (Dreywood, 1946). The contents of titratable acid, vitamin C, and nitrate were determined following national standards of GB 12293–90 (SBQTS 1990), GB 5009.86–2016 (NHFPC 2016), and GB 5009.33–2016 (NHFPC and FDA 2016), respectively.

### 2.3. DNA extraction, PCR amplification, and high-throughput sequencing

Soil samples (separately in two depths, 0–20 cm and 20–40 cm) were taken at six spots in root zone that were 1.5 m away from west side of three trees (in one block) and mixed together as one replication of each treatment on July 19, 2021. The collected samples were then transported to the lab in a box with ice packs, sieved (2 mm) and sent to Majorbio Bio-Pharm Technology Co. Ltd. with dry ice. Total bacterial community genomic DNA was extracted from 0.5 g fresh soil samples of surface (0–20 cm) and subsurface soils (20–40 cm). A pair of primers 338F (5’-ACTCCTACGGGAGGCAGCAG-3′) and 806R (5’-GGACTACHVGGGTWTCTAAT-3′) was used for bacterial 16S rDNA gene amplification in the hypervariable V3-V4 region. The polymerase chain reaction (PCR) was conducted with an ABI GeneAmp® 9,700 PCR thermocycler (ABI, CA, United States), with amplification conditions and PCR reaction mixture that can be found in Sun et al. (2022). PCR products were quantified using Quantus™ Fluorometer (Promega, United States) after purification. Purified amplicons were pooled in equimolar and paired-end sequenced on an Illumina MiSeq PE300 platform (Illumina, San Diego, United States) according to the standard protocols by Majorbio Bio-Pharm Technology Co. Ltd. (Shanghai, China). The obtained gene sequences from Illumina platform are deposited in the National Center for Biotechnology Information (NCBI) Sequence Read Archive with the accession number PRJNA835607.

The sequencing data were trimmed for barcodes and primers. Low-quality reads less than Q20 were removed (Magoč and Salzberg, 2011). The 18 test samples had a total number of 958,788 effective sequences with an average length of 417 bp. High-quality sequences were clustered into operational taxonomic units (OTUs) with a 97% similarity using UPARSE (^1^version 7.1). RDP classifier (^2^version 2.2) was used for comparison of the representative sequence of each OUT to the database Silva (Release115^3^) with a confidence threshold of 0.7 (Wang et al., 2007).

### 2.4. Data analysis

Analysis of variance (ANOVA) was conducted for foliar spectral traits (NDVI, GI, OSAVI, PRI, NPCI, MCARI, and SIPI), fruit attributes (single fruit weight, transverse diameter, vertical diameter, nitrate content, vitamin C, soluble sugar, titratable acid, and total soluble solid), and alpha diversity estimates (Sobs, Shannon, Simpson, ACE, and Chao1) as a function of three treatments at different soil depths using the PROC ANOVA procedure in the SAS statistical software package (SAS Institute Inc., Cary NC. United States). Shannon and Simpson estimate bacterial community diversity while ACE and Chao1 estimate bacterial community richness. Alpha-diversity metrics were assessed using Mothur (^4^version 1.31.2). Means were compared using the least significant difference test at the 5% probability level. The 30 most abundant genera at both soil depths of all treatments were compared *via* phyla stacked column diagram analysis in Origin 2021 (OriginLab Corporation, Northampton, MA, United States).

Bacterial genera in this study were firstly divided in identified or unidentified genera. The identified genera were then defined as abundant (>1%) or rare (<1%) genera. Beta-diversity of all treatments was calculated with the Bray–Curtis dissimilarity metric and visualized in a Principal Coordinates Analysis (PCoA) plot using R version 3.3.1 (Linux-GNU). Spearman correlation analysis was conducted between alpha diversity estimates (fruit attributes) and foliar spectral indexes and demonstrated in a heatmap *via* OriginLab. A redundancy analysis (RDA) was performed for relationships between foliar spectral indexes and fruit attributes in CANOCO 5.0. The manual forward-selection procedure was used to determine significance of soil characteristic variables (*p* < 0.05) using a Monte Carlo test with 499 permutations. Linear regression model was conducted to analyze the relations between foliar spectral indexes and alpha diversity estimates in OriginLab. Clustered heatmap analysis of the relations between foliar spectral indexes and the 30 most abundant genera were conducted using the pheatmap package in R (version 3.3.1, Linux-GNU).

Independent structural equation models (SEMs) were constructed at both soil depths, based on known effects and relationships among the drivers of plant productivity. Since some of the variables introduced were not normally distributed, the probability of a path coefficient differs from zero was tested using bootstrap resampling. Thus, data are randomly sampled with replacement to arrive at estimates of standard errors that are empirically associated with the distribution of the data found in the samples (Grace et al., 2010). For the structural model, we used the acceptable fit by Hair et al. (2019): Collinearity Statistics (VIF ≥ 5, probable critical collinearity; 5 > VIF ≥ 3, possible collinearity; VIF < 3, ideally show). The best fit SEM was accomplished *via* T Statistics (T > 1.96), *p* values (*p* < 0.05), root mean square error of approximation (SRMR; SRMR <0.08), and Comparative fit index (d_ULS < 0.95 and d_G < 0.95) (Hair et al., 2019). All the SEM analyses were used SmartPLS (^5^ version 3.0).

## 3. Results and discussion

### 3.1. Linkage between foliar spectral traits and plant productivity characteristics

A previous study reported that spectral functional diversity of plant community explained 51% of the total variation of plant productivity (*r*^2^ = 0.51, *p* < 0.001, Schweiger et al., 2018). This linkage between foliar spectral traits, functional, and evolutionary divergence provides the rationale for using spectral profiles in estimating biodiversity (Maherali and Klironomos, 2007; Cadotte et al., 2008; Hector et al., 2010). However, it is still challenging to choose spectral functional diversity indexes to capture the productivity of plant community that is location and time specific (Schweiger et al., 2018). Ultimately, the effects of biodiversity on plant community are a result of individual variation (Bolnick et al., 2011; Violle et al., 2012). Considering foliar spectral traits can be acquired relatively rapid on a regular basis than traditional protocols, we proposed to employ foliar spectral traits as indicators for plant productivity in orchard ecosystem.

**Figure 1:**
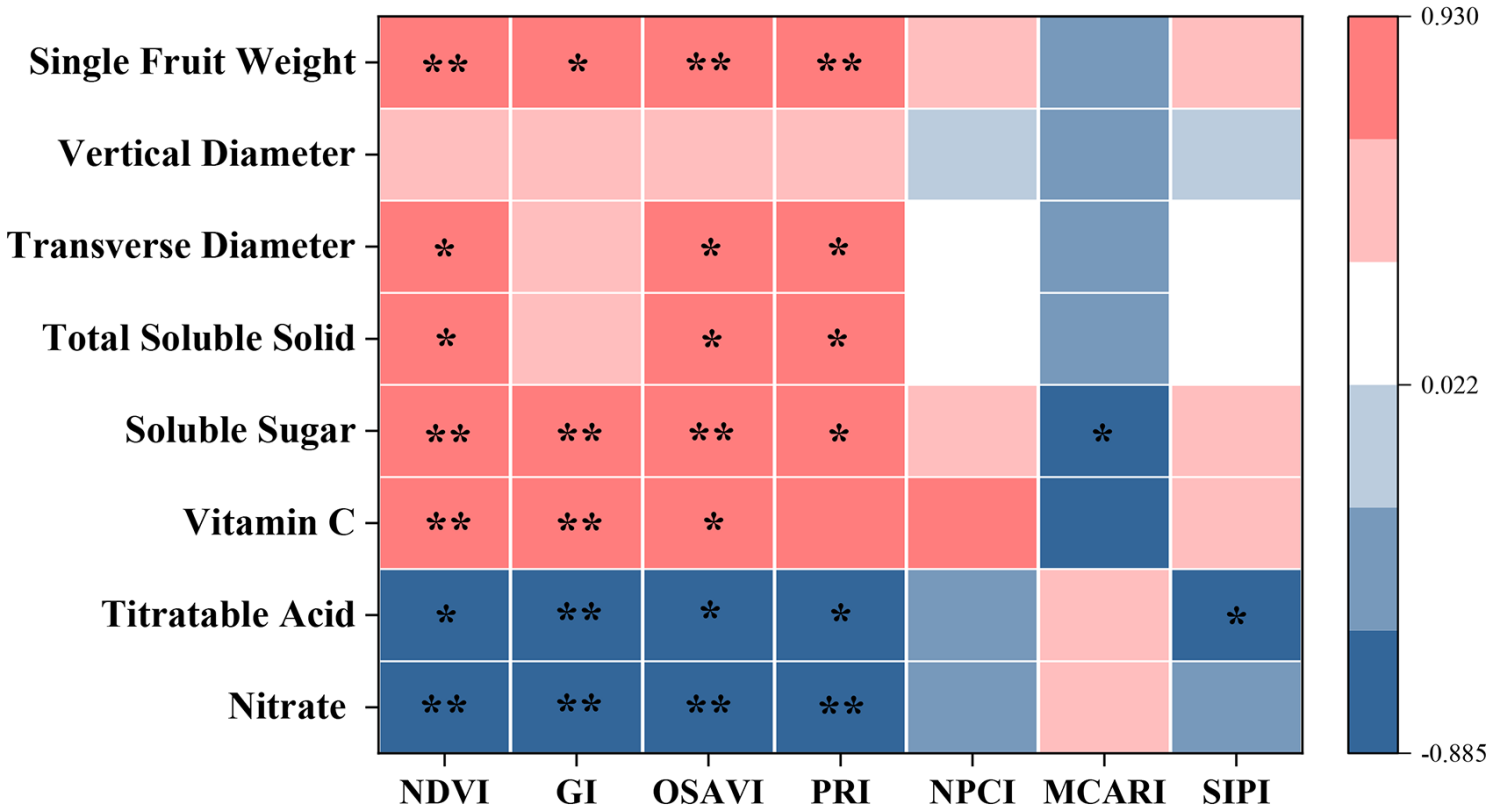
Spearman correlation analysis between foliar spectral indexes and fruit attributes. * indicates significant correlation (*p* < 0.05), while ** indicates extremely significant correlation (*p* < 0.01).

In this study, RERP had positive effects on foliar spectral traits, fruit yield and quality (p < 0.05), with T1 a more efficient approach than T2 (Supplementary Tables S3, S4). Spearman correlation analysis revealed that selected spectral indexes of NDVI, GI, OSAVI, and PRI were significantly correlated to yield (single fruit weight) and quality (nitrate titratable acid, Vc, soluble sugar, and total soluble solid contents) as shown in Figure 1. Previous studies reported similar results of foliar spectral traits in assessing plant attributes (productivity) in other semi-arid ecosystems. Vegetation indexes, such as NDVI, GI, and OSAVI, reflected information of leaf area index and chlorophyll content, and were often used in inversion of vegetation coverage and growth status (Li et al., 2009; Ferna et al., 2018; Yang et al., 2020). NDVI and OSAVI estimated green biomass and vegetative coverage in grass ecosystem (Ferna et al., 2018), while PRI estimates instant light use efficiency at leaf scale, which was used to track changes of photoprotective pigment pools and capture eco-hydrologic sensitivity of a mixed conifer forest (Yang et al., 2020). Daily PRI measurements could be used to track changes of photoprotective pigment pools as plants respond to seasonal environment change. OSAVI introduced the factor of bare land in the calculation, which was in accordance with the increased bare land area from agricultural practices (such as weeding and pruning) for better growing conditions and higher yield in the orchard (Shu, 2003). Meanwhile, these agricultural practices affected fruit availability of photosynthetically active radiation, and consequently fruit sensorial and nutritional quality (Gullo et al., 2014).

**Figure 2:**
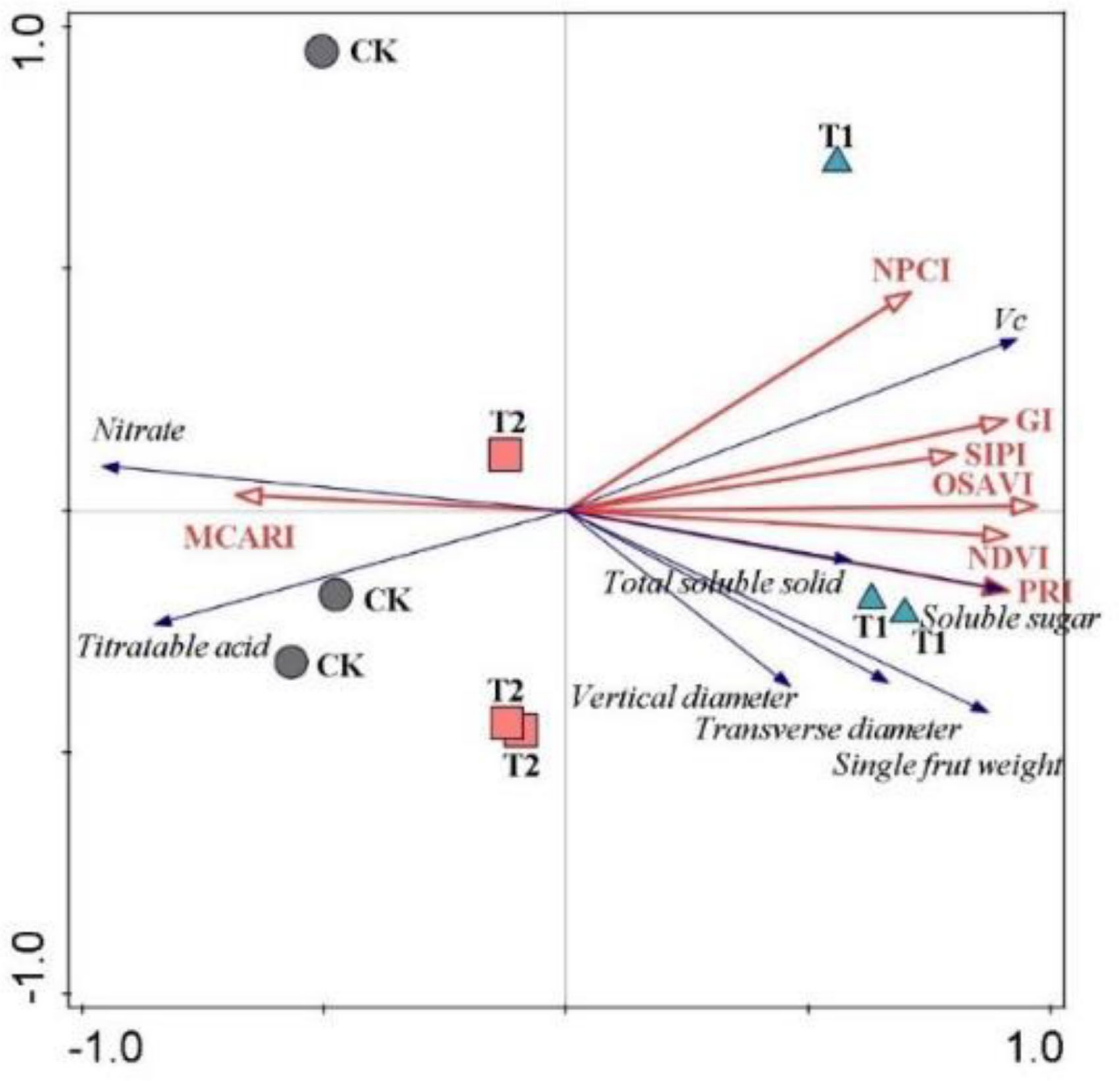
Redundancy analysis of relationships between foliar spectral indexes and fruit attributes.

**Figure 3:**
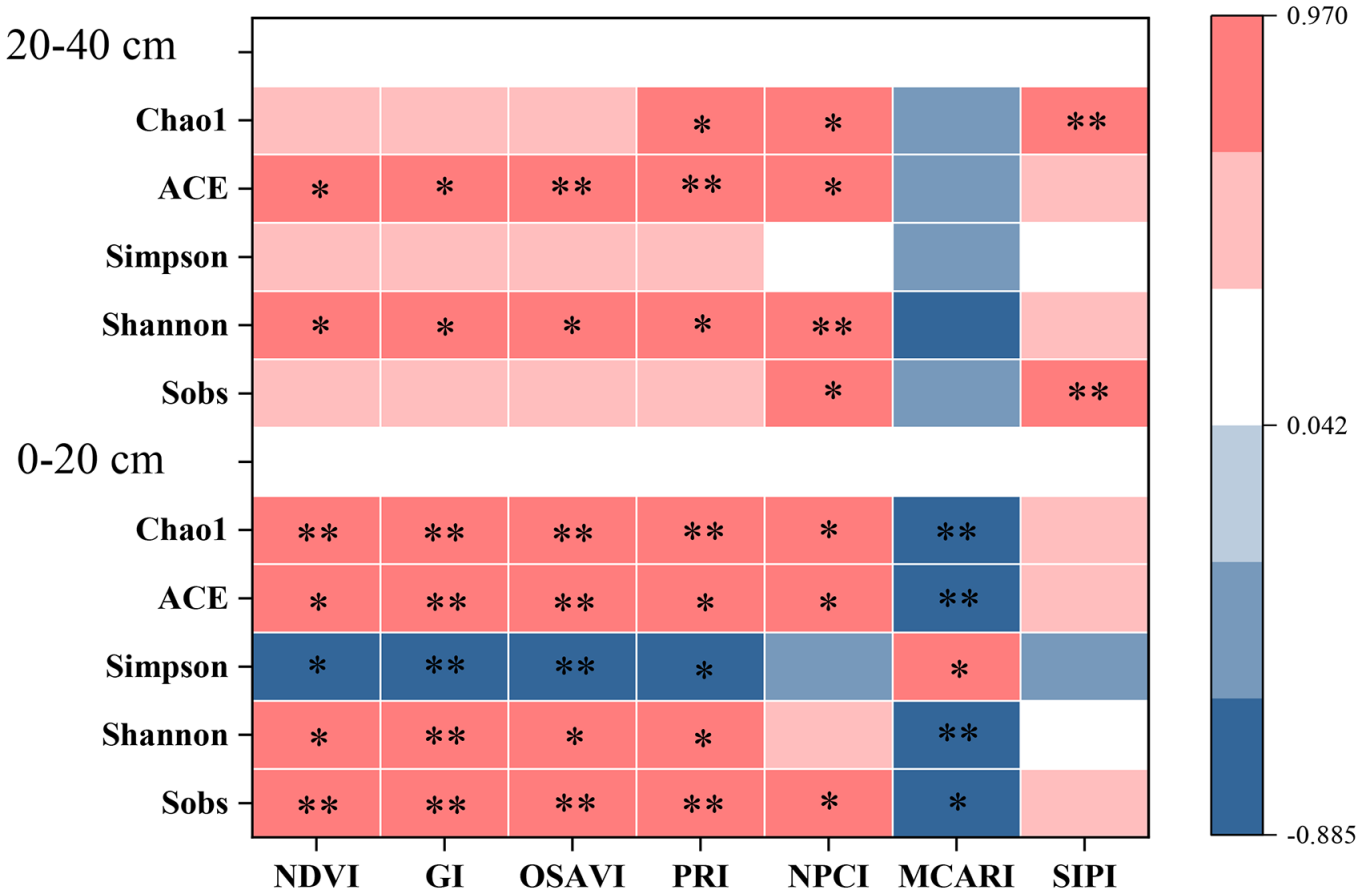
Spearman correlation analysis between spectral indexes and alpha diversity of bacterial community in surface and subsurface soils. * indicates significant correlation (*p* < 0.05), while ** indicates extremely significant correlation (*p* < 0.01).

RDA analysis further revealed a relationship between spectral traits and plant productivity (Figure 2). T1 was distinct from CK and T2, indicating a profound effect of RERP with soil conditioner (T1) on fruit yield and quality (Figure 3). The seven spectral indexes accounted for 94.3% in the first two constrained axes (axis 1 and 2 explained 83.15 and 11.15% of the variance, respectively). OSAVI was the only index that greatly contributed to the total variance (78.8%, *p* = 0.002). Spectral reflectance profiles are continuous representations of the interaction between electromagnetic radiation and matter across a range of wavelengths. In visible spectrum (400-700 nm), light is predominantly absorbed by leaf pigments (Ustin et al., 2009; Sul et al., 2013). Schweiger et al. (2018) also proved that spectral diversity (400–900 nm) was as predictive of ecosystem function as functional, phylogenetic or taxonomic diversity. Foliar spectral indexes were therefore used to assess biochemical traits that are significant in understanding plant growth and physiological status (Díaz and Cabido, 2001; Lavorel and Garnier, 2002; Wright et al., 2004; Asner et al., 2011; Lavorel et al., 2011). The results above suggested that plant productivity embodied in fruit yield and quality in orchard ecosystem could reflect in foliar traits and be predicted by foliar spectral indexes.

### 3.2. Linkage between foliar spectral traits and soil bacterial alpha diversity

Individuals with distinct ecological phenotypes within a plant community may influence soil microbial community (Scheibe et al., 2015; Gehring et al., 2016). In fact, plant ecological phenotype was not only displayed as distinct physical structures (plant organ, tissue, and cell), but also reflected in the quantizable phenotypic parameters related to plant physiology and biochemistry (Ustin et al., 2009; Ustin, 2013; Scheres and Van Der Putten, 2017), such as foliar spectral traits (Schweiger et al., 2018). Although leaf and canopy optical data from imaging spectroscopy were incorporate into ecological studies (Asner et al., 2011; Doughty et al., 2017), foliar spectral traits were seldomly studied in association with belowground microbial diversity.

In the present study, RERP (T1 in particular) had positive effects on bacterial alpha diversity that were soil depth dependent (Supplementary Table S5). T1 significantly increased bacterial diversity (Shannon) and richness (ACE and Chao1) in both surface and subsurface soils, mainly due to increased nutrient availability (N, K, Ca, Mg, and Zn), soil organic matter content, water holding capacity, soil porosity, and decreased soil bulk density (Sun et al., 2022). Stronger correlations between foliar spectral indexes and bacterial alpha diversity were observed in surface soil than in subsurface soil (Figure 3). Each foliar spectral index was significantly correlated to at least one bacterial diversity and richness estimates. NDVI, GI, and OSAVI were significantly correlated to all bacterial diversity and richness estimates in surface soil, but only to ACE and Shannon in subsurface soil. OSAVI had the highest correlation coefficients with alpha diversity estimate, such as Sobs, ACE, and Chao1 in surface soil and ACE and Shannon in subsurface soil. PRI and NPCI, relevant to leaf photosynthesis were correlated to both diversity and richness estimates at both soil depths. Although MCARI and SIPI were pertinent to foliar pigments, MCARI was only significantly correlated to alpha diversity in surface soil, while SIPI was better indicative of Sobs and Chao1 in subsurface soil.

**Figure 4:**
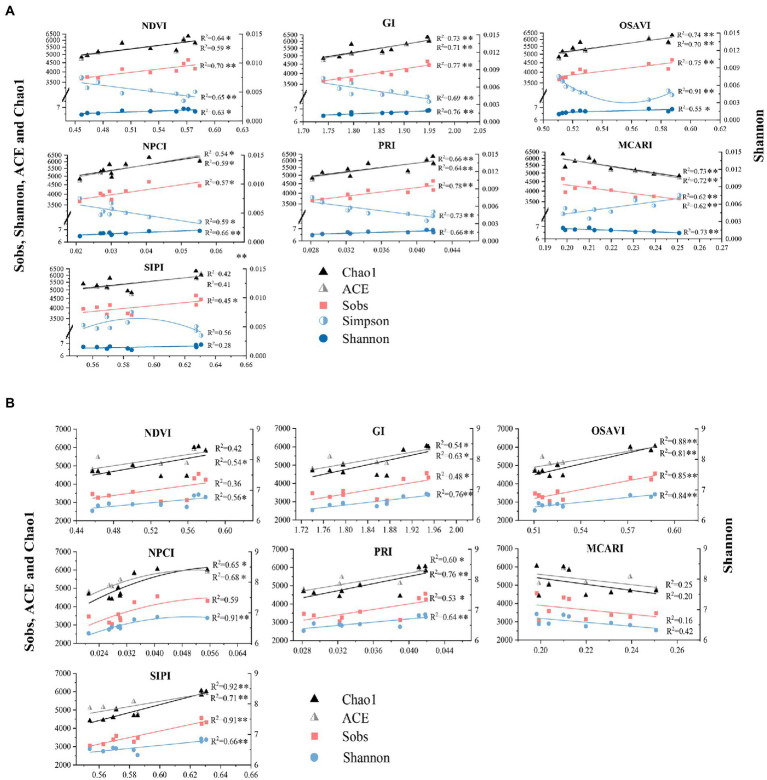
Linear regression analysis between foliar spectral indexes and alpha diversity estimates of bacterial community in surface **(A)** and subsurface **(B)** soils. * indicates significant correlation (*p* < 0.05), while ** indicates extremely significant correlation (*p* < 0.01).

Correlation analysis revealed that bacterial alpha diversity estimates were correlated with foliar spectral indexes to different extents. It is not clear whether the relations could be quantified by reliable models for prediction of bacterial diversity. Linear regression model was then employed to quantify the association between soil bacterial diversity and foliar spectral indexes (Figures 4A,B). Regression analysis results suggested that foliar spectral indexes could be adopted to predict bacterial alpha diversity. The relationships between foliar spectral indexes and bacterial diversity estimates varied by soil depths and the specific index (estimate). OSAVI, GI, and MCARI in surface soil, and OSAVI, PRI, GI, and SIPI in subsurface soil could directly explain 53–92% variance of bacterial alpha diversity. Foliar spectral indexes were more indicative of bacterial richness estimates than diversity estimates in general. Similarly, Cavender-Bares et al. (2021) reported remotely sensed vegetation cover was positively correlated with Simpson in prairie ecosystem with high productivity in Wood River, while Aji et al. (2021) reported a weak correlation between a large scale NDVI and soil microbial biomass in natural ecosystems (including lake area and protested forests) with minimum human disturbance. Generally, spectral indexes were more indicative of bacterial richness estimates than diversity estimates, and in subsurface soil than surface soil for all bacterial diversity estimates, except for Simpson. To date, few studies have been conducted on the association between specific spectral index and bacterial alpha diversity. In last decades, plant productivity was affected by declined microbial diversity from human activities and climate changes. This has triggered concerns of soil microbial diversity loss may impair key ecosystem functions with potential consequences on plant productivity (Cadotte, 2017; Schweiger et al., 2018; Chen et al., 2019). Therefore, avoiding or alleviating the loss of soil microbial diversity is critical in ecosystem (Chen Q. et al., 2020).

In this study, we draw the above conclusions based on the experimental orchard at the sampling time. Due to seasonal shifts in alpha diversity of soil bacterial community (Shi et al., 2015; Wagner et al., 2016), and in foliar spectral traits of deciduous trees in temperate arid and semiarid regions (Pederson et al., 2014), it is too early to decide if this quantified relations between foliar spectral traits and bacterial diversity can be applied at other growing stages of orchard ecosystem or to other ecosystems with the same spectral indexes at the same soil depths. Deeper understanding of specialized spectral indexes for further investigation of bacterial community variation is needed.

### 3.3. Linkage between foliar spectral traits and soil bacterial beta diversity

**Figure 5:**
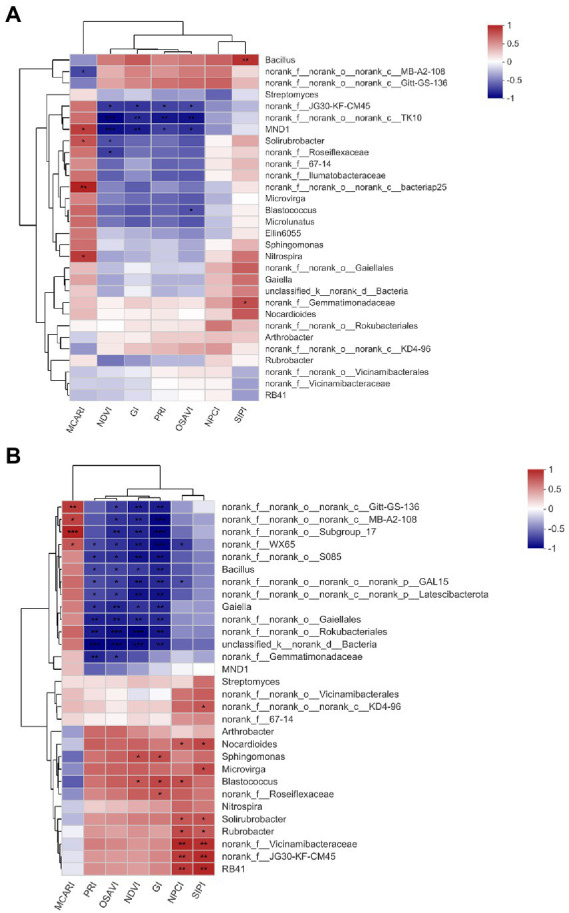
Clustered heat map analysis of the relationships between foliar spectral indexes and the 30 most abundant genera in surface **(A)** and subsurface **(B)** soils. * indicates significant correlation (*p* < 0.05), while ** indicates extremely significant correlation (*p* < 0.01).

Soil bacterial assembly plays a more important role in driving soil bacterial and ecological functioning than bacterial alpha diversity (Bever et al., 2012; Bardgett and Van der Putten, 2014; Leff et al., 2018), which drove us into exploring whether and which foliar spectral traits could be employed as indicators of certain bacterial genera. RERP (both T1 and T2) significantly affected the bacterial composition on genus level in surface soil and subsurface soil (Supplementary Figure S1). Improved soil properties from RERP enriched certain genera (such as *Blastococcus*, *Bacillus*, *Sphingomonas*, and *Solirubrobacter*) in relation to increased soil nutrient content, organic matter, porosity, and water content (Sun et al., 2022). PCoA revealed larger discrepancy of bacterial community of three treatments in subsurface soil than in surface soil on genus level (Supplementary Figures S2A,B). In subsurface soil, 76.73% of the total variance was explained by the first two components. Samples of T1 were grouped together and distinct from samples of CK and T2. According to spearman correlation analysis (Figures 5A,B), more genera were in significant correlations with foliar spectral indexes in subsurface soil than in surface soil, possibly due to the frequent agricultural practices that have reduced differences between treatments in the orchard, or the more bacterial functional redundancy in surface soil. *MND1* in surface soil, and nine genera in subsurface soil (*Bacillus*, *Gaiella*, *Nocardioides*, *Sphingomonas*, *Microvirga*, *Blastococcus*, *Solirubrobacter*, *Rubrobacter*, and *RB41*) were significantly correlated to at least one foliar spectral index (NDVI, GI, OSAVI, and PRI in particular). Furthermore, correlations between the same foliar spectral indexes and soil bacteria genera were interacted with soil depth. Some genera were only in significant relations with certain foliar spectral indexes in one soil layer, such as *MND1* in surface soil (absolute value of correlation coefficient: 0.72–0.90, *p* < 0.05), and *Gaiella*, *Nocardioides,* and *Rubrobacter* in subsurface soil (absolute value of correlation coefficient: 0.67–0.82, p < 0.05).Some genera had opposite and enhanced relations with foliar spectral indexes at these two soil layers. For instance, *Bacillus* was in positive relation with SIPI in surface soil (correlation coefficient: 0.80, p < 0.05), but in negative relations with NDVI, GI, PRI, and OSAVI in subsurface soil (correlation coefficient: −0.7 to −0.82, p < 0.05). *Blastococcus* was in negative correlation with OSAVI (correlation coefficient: −0.67, *p* < 0.05), but in positive relations with NDVI, GI, and NPCI (correlation coefficient: 0.68 to 0.75, *p* < 0.05). *Gaiella* (reduce nitrate to nitrite) were involved in N cycle, which could affect soil N content and plant growth that further reflect on foliar spectral characteristics (Asner et al., 2011; Sul et al., 2013; Sebin et al., 2014). MND1 and *Nocardioides* were relevant to C and N nutrient cycling (Sul et al., 2013; Zhang et al., 2022) while *Solirubrobacter* were reported to affect sulfur availability for peach plants (Liu et al., 2022). *Microvirga* belong to α-proteobacterial that favored for nutritious soil (Sul et al., 2013). *Blastococcus* degrade organic material through proteolysis (Chen et al., 2016; Duan et al., 2019). *Sphingomonas* have been reported as plant growth promoting bacteria (Asaf et al., 2020; Chen L. et al., 2020) and was active in the biocontrol of pathogenic bacteria (Innerebner et al., 2011). According to previous studies, these bacterial genera in strong correlation with foliar spectral traits were those promoted soil nutrients (such as C, N, P, K, and S) cycling and uptake. This was supported by previous conclusion that foliar spectral profiles can reflect plant traits (including leaf structure, and the contents of pigments, nutrients and water) that were key to resource acquisition and stress tolerance (Sebin et al., 2014; Schweiger et al., 2018). Among these bacteria, genera with greater abundance discrepancy between treatments were in stronger correlations with foliar spectral indexes, indicating that leaf and canopy optical data were responsive to structural variance of bacterial community. It was speculated that these genera might have greater impact on aboveground plant.

However, we only investigated the 30 most abundant genera with relative abundances >1%. Certain unidentified or abundances <1% genera may have played an over-proportional role in biological processes, and being the major drive for bacterial community multifunctionality (Chen L. et al., 2020). Incomplete knowledge of these bacteria hindered further illustration of their function and contribution to plant productivity. Functional redundancy may have covered the distinction of soil microbial community, and compensated ecological functioning and plant productivity of the orchard ecosystem, which could have interfered the investigation on the relations between bacterial components and foliar spectral traits. Under the assumption that these genera in significant correlation with foliar spectral traits were key to ecosystem functioning, further investigation is in need on if exogenous addition of these bacteria in bio-organic fertilizer could improve plant productivity and ecological functioning in fruit orchards (Gottel et al., 2011; Lundberg et al., 2012; Wagner et al., 2016).

### 3.4. Structural equation models between foliar spectral traits and soil bacterial community

**Figure 6:**
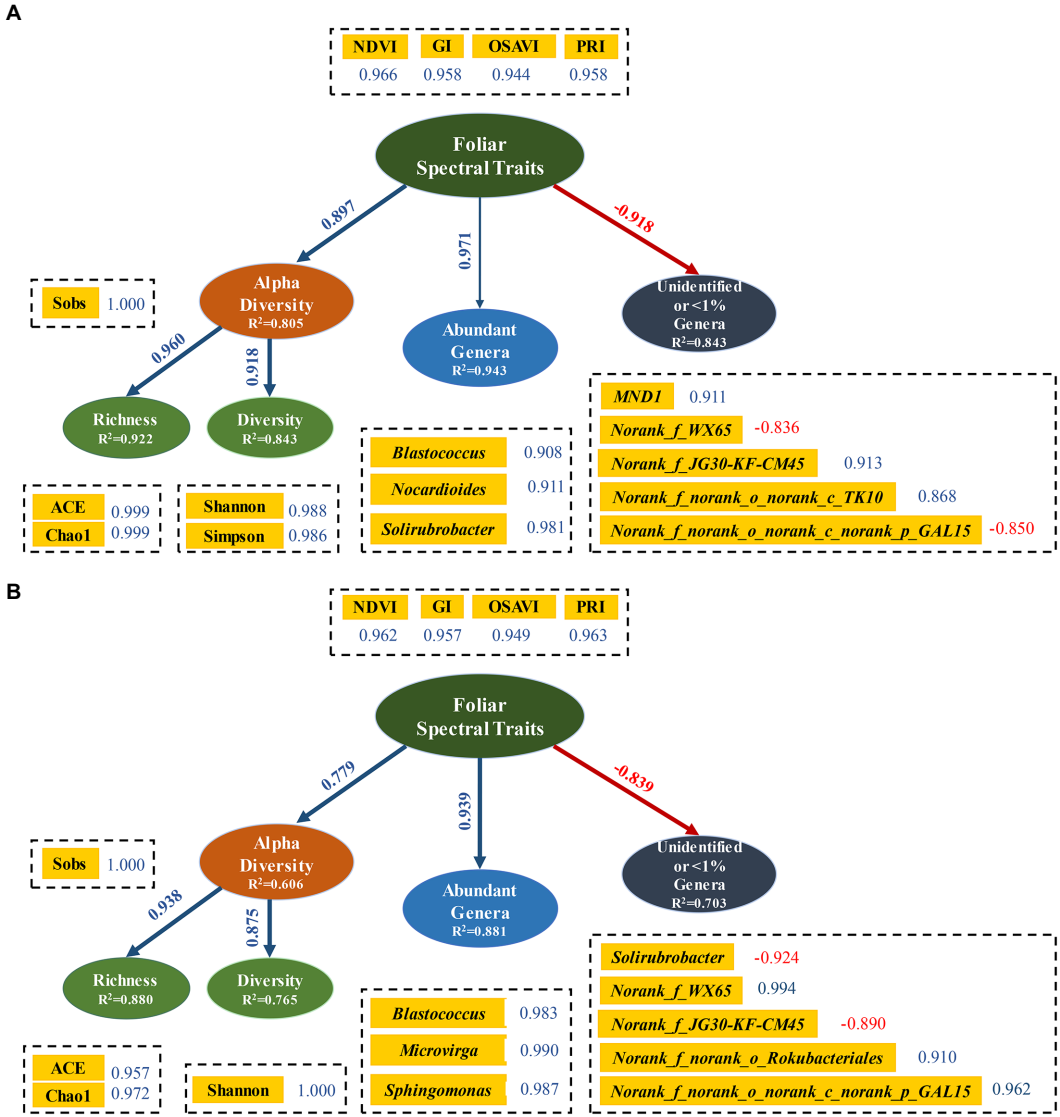
Structural equation models of relationships among foliar spectral traits, alpha diversity, abundant genera and rare genera in surface **(A)** and subsurface **(B)** soils. Blue lines indicate positive correlations. Red lines and numbers indicate negative correlations. Rare genera were with relative abundances <1%. Relationship between foliar spectral traits and abundant genera was not significant (*p* = 0.086), indicating with a thin line, while all other relations were significant (*p* < 0.05) with thicker lines.

Structural equation modeling (SEM) analyzes the complex networks of causal relationships among multiple variables, which has been increasingly adopted in ecological sciences in the last decade (Grace et al., 2010; Eisenhauer et al., 2015). Four similar latent variables were employed in both soil layers, with three of them (bacterial diversity, abundant genera and unidentified or < 1% genera) had soil-depth dependent indicators (Figures 6A,B). NDVI, PRI, GI, and OSAVI were best indicators of foliar spectral traits to detect bacterial diversity. Similarly, Delgado-Baquerizo et al. (2017) used NDVI as indicator of plant productivity, while Ferna et al. (2018) suggested NDVI and OSAVI as indicators of green biomass and coverage in a semi-arid rangeland. All variables were explained with different proportion of variance by the selected indicators. Generally, variables had higher R2 value in surface soil than in subsurface soil. This is possible due to the interference from frequent agricultural practices which resulted in a less effective approach of RERP in topsoil. Meanwhile, correlation coefficients between indicators and variables were higher in surface soil, which was in accordance with the higher bacterial diversity in surface soil (Delgado-Baquerizo et al., 2017). Foliar spectral traits were in negative association with unidentified or < 1% genera, and in positive associations with all other variables. Furthermore, all path coefficients were extremely significant, except for association between abundant genera and foliar spectral traits in surface soil. This is likely due to the non-significant impacts of abundant genus in topsoil on plant growth, or functional redundancy from enriched abundant genera in topsoil, or the limited soil samples taken from the orchard.

Although experimental and observational databases in ecological studies are often complex, non-randomly distributed, and have spatial and temporal constraints (i.e., potential autocorrelations), SEM in this study indicated that foliar spectral traits (measured for peach productivity) were effective in predicting soil bacterial diversity and key genera. Latent variables, indicators and pathway coefficient of SEM in semi-arid peach orchard lay groundwork for further forecasting of bacterial diversity and structure in surface and subsurface soils. Environment, climate, and soil properties would all affect bacterial community and foliar spectral traits on a large scale. In this study, we focused on using foliar spectral traits to predict bacterial diversity at low cost, by using RERP to alter soil properties and obtain differentiated soil bacterial community (Sun et al., 2022). Since we conducted the field experiment in a peach orchard, the environmental and climatic factors were the same among treatments in this small scale. Although foliar spectral traits and soil microorganisms have obvious temporal dynamics, we selected the maturity period of experimental peach (July) as the critical timing for foliar spectral traits to represent productivity, since peach trees bear fruit only once a year in Beijing and the productivity of fruit trees is mainly reflected by fruit yield and quality. Spectral traits of typical leaves could then be used to characterize bacterial diversity and taxonomy. Of course, sampling on large scale with temporal dynamic are needed in order to obtain the general relationships between foliar spectra traits and soil bacteria community.

Generally, soil microbes were constrained by abiotic factors at large temporal and spatial scales, and by biotic factors at regional scale (Wang et al., 2015; Zhang et al., 2020). Ji et al. (2011) reported that cultivar, rather than tree age, played an important role in dominant bacteria community of rhizosphere soil in peach orchards in Shanghai. This has led us to the question: if the quantified linkage between foliar spectral indexes and soil bacterial community is applicable to other peach cultivar or to the same cultivar at different tree ages. Furthermore, can the quantified linkage be applied to other orchard ecosystems? Shao et al. (2020) reported eight deciduous fruit trees with different levels of metabolism and functional diversity of the soil bacterial community. Schweiger et al. (2017) also argued that spectral detectability of plant life/growth forms was dependent on the studied ecosystem and plant community. If this linkage can be extended to other fruit orchards, we could provide new insights into the mechanisms of optimizing plant productivity and maintaining plant community structure.

Hyperspectral measurements acquired from the method of airborne hyperspectral imaging in combination with high precision GNSS/IMU have improved spatial and spectral resolution to a centimeter level accuracy, which revealed messages that could not recognized by RGB or multi-spectral imaging. Recently, plant functional diversity was reported better indicated by spectral indexes in red edge, near infrared, shortwave infrared regions and other hyperspectral images, rather than in visible region (Asner et al., 2017; Schneider et al., 2017; Schweiger et al., 2018; Ma et al., 2019; Cavender-Bares et al., 2021, 2022). In future research, foliar spectral characteristics in near and far infrared region could be further looked into for the elucidation of broadened ecological linkages between foliar spectral characteristics and soil bacterial community in orchard ecosystem.

## 4. Conclusion

Foliar spectral traits are indicative of plant productivity and could be used to predict soil bacterial diversity in peach orchard. NDVI, GI, PRI, and in particularly OSAVI were selected as appropriate indicators of plant productivity. SEM revealed quantified linkages between foliar spectral indexes and bacterial richness, bacterial diversity, abundant genera, and unidentified or < 1% genera in both surface and subsurface soils. The results of this study enabled deeper understanding of the mechanisms in responses and adaptations of fruit trees to agricultural practices in the orchard ecosystem. Foliar spectral indexes could efficiently monitor soil bacterial diversity and functionality at low cost, with an ultimate goal of regulating plant physiological function by adjusting agricultural practices and the consequent microbial community accordingly.

However, additional information is in need on if the reliability of foliar spectral traits in predicting soil microbial community would be affected by plant species, environmental conditions and agricultural practices. A new concept of adopting foliar spectral traits as plant traits has simplified the research in exploring the associations between aboveground plant traits and belowground biodiversity at the scale of orchard ecosystem. With the scalability and repeatability of valid spectral indexes, this research could be scaled up to community, field, regional, and even terrestrial levels, to better realize the forecasting and regulating of aboveground and underground functionality in agricultural system, and further trade-ones of ecological and economical function in agroecological system.

## Data availability statement

The datasets presented in this study can be found in online repositories. The names of the repository/repositories and accession number(s) can be found in the article/Supplementary material.

## Author contributions

NS: data curation, formal analysis, funding acquisition, writing-original draft, and writing-review and editing. WZ: conceptualization, data curation, and writing-review and editing. SL: writing-review and editing. HL: conceptualization, data curation, formal analysis, methodology, funding acquisition, writing-original draft, and writing-review and editing. All authors contributed to the article and approved the submitted version.

## Funding

This work was supported by Beijing Science and Technology Project of Beijing Municipal & Technology Commission (Z191100004019001), and Youth Research Fund of Beijing Academy of Agriculture and Forestry Sciences (QNJJ202202), and Beijing Leisure Agriculture Innovation Consortium (BJLA-G09).

## Conflict of interest

The authors declare that the research was conducted in the absence of any commercial or financial relationships that could be construed as a potential conflict of interest.

## Publisher’s note

All claims expressed in this article are solely those of the authors and do not necessarily represent those of their affiliated organizations, or those of the publisher, the editors and the reviewers. Any product that may be evaluated in this article, or claim that may be made by its manufacturer, is not guaranteed or endorsed by the publisher.

## Footnotes

^1^ http://drive5.com/uparse/

^2^ http://rdp.cme.msu.edu/

^3^ http://www.arb-silva.de

^4^ http://www.mothur.org/

^5^ http://www.smartpls.com
