# Supplementary material for: Is foliar spectrum predictive of belowground bacterial diversity? A case study in a peach orchard
Source: Front Microbiol. 2023 Feb 24;14:1129042. doi: 10.3389/fmicb.2023.1129042 (PMC9998905; doi:10.3389/fmicb.2023.1129042)
Supplement: Supplementary file 1 [file Data_Sheet_1.docx]

Supplementary Material

# Supplementary Figures and Tables

## Supplementary Figures

**Supplementary Figure S1**. Bacterial composition (30 most abundant genus)as affected by RERP in surface (a) and subsurface (b) soils


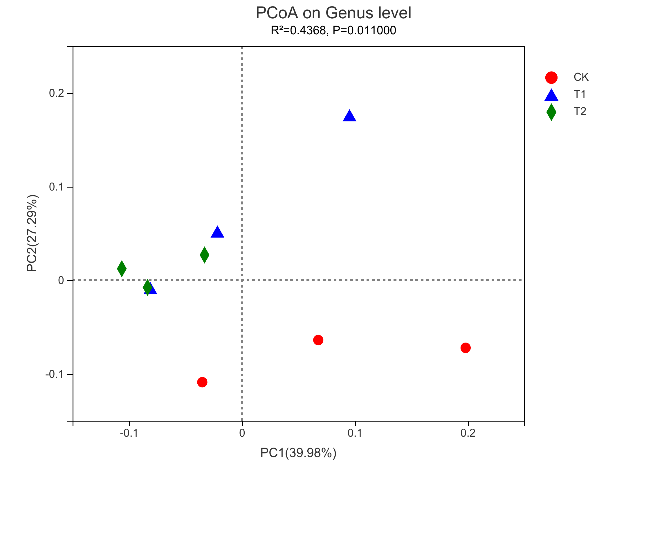

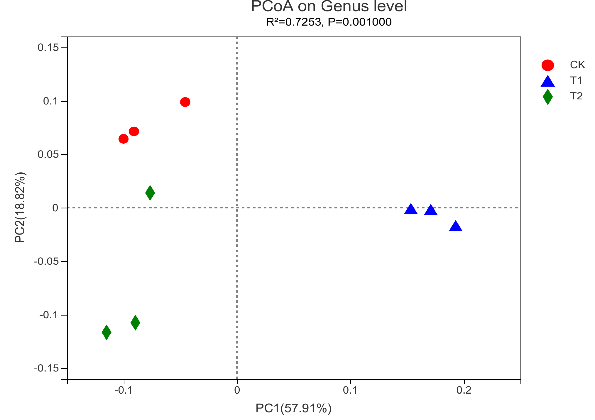


b

a

**Supplementary Figure S2**. Principal coordinates analysis of soil bacterial community on genus level of all three treatments in surface (a) and subsurface (b) soils

## Supplementary Tables

**Supplementary** **Table S1**. Pre-treatment soil properties at different soil depths in the experimental peach orchard

| Soil Depth | Alkali-hydrolysable N | Available P | Available K |  | Total N | Total P | Total K |  | Soil Organic Matter | CEC |  | Soluble Ca | Soluble Mg | Soluble K | Soluble Na |
| --- | --- | --- | --- | --- | --- | --- | --- | --- | --- | --- | --- | --- | --- | --- | --- |
| cm | mg kg^-1^ | | |  | % | | |  | g kg^-1^ |  |  | cmol kg^-1^ | | | |
| 0-20 | 105 | 81.9 | 457.67 |  | 0.17 | 0.10 | 2.03 |  | 22.8 | 9.42 |  | 0.31 | 0.12 | 0.06 | 0.11 |
| 20-40 | 90.47 | 19.77 | 133.33 |  | 0.11 | 0.06 | 1.98 |  | 18.9 | 9.35 |  | 0.24 | 0.06 | 0.01 | 0.18 |

| Spectral Index | Equation | Reference | Implication and Application |
| --- | --- | --- | --- |
| NDVI | (R_800_-R_680_)/(R_800_+R_680_) | Rouse et al. (1974) | NDVI could quantify vegetation and often indicates vegetation amount (relative biomass). |
| GI | R_554_/R_667_ | Zarco-Zejada et al. (2005) | GI is known as the ratio vegetation index, reflecting vegetation coverage and growth status. It is suitable to monitor vegetation with vigorous growth and high vegetation coverage. |
| OSAVI | (1+0.16)*(R790-R670)/(R790-R670+0.16) | Rondeaux et al. (1996) | OSAVI mainly takes soil factors into consideration on the basis of NDVI. It excludes influences from soil and suitable to reflect chlorophyll content in the early growth stage or under low vegetation density. |
| PRI | (R_531_-R_570_)/(R_531_+R_570_) | Gamon et al. (1992) | PRI is the normalized vegetation index related to canopy light use efficiency. It is usually used to study canopy light use efficiency and stress response. |
| NPCI | (R_680_-R_430_)/(R_680_+R_430_) | Peñuelas et al. (1994) | NPCI is a normalized chlorophyll index, which is used to estimate foliar pigment content. It can avoid the absorption region overlapping from different foliar pigments, and the influences on absorbance from leaf and canopy structure. |
| MCARI | [(R_700_-R_670_)-0.2*(R_790_-R_550_)]*(R_700_/R_670_) | Daughtry et al. (2000) | MCARI can effectively reduce the influence of photosynthetic active radiation changes caused by non-photosynthetic substances in the canopy. Yet it is easily affected by background soil reflectance. |
| SIPI | (R_790_-R_450_)/(R_790_+R_650_) | Peñuelas et al. (1995) | SIPI can minimize the influence from canopy structural variables (such as LAI), well characterize mesophyll structure, and indicate changes in the ratio of carotenoid to chlorophyll. It can be applied to vegetation health monitoring, plant physiological stress detection, crop production and yield analysis. |

**Supplementary** **Table** **S2**. Equations of foliar spectral indexes adopted in this **study**

**Supplementary** **Table** **S3**. Analysis of variance for effects of RERP on foliar spectral traits in the peach orchard

| Treatment | NDVI | GI | OSAVI | PRI | NPCI | MCARI | SIPI |
| --- | --- | --- | --- | --- | --- | --- | --- |
| CK | 0.46 b | 1.77 c | 0.51 b | 0.030 b | 0.027 b | 0.24 a | 0.58 b |
| T1 | 0.57 a | 1.93 a | 0.58 a | 0.042 a | 0.043 a | 0.21 b | 0.63 a |
| T2 | 0.53 a | 1.84 b | 0.52 b | 0.035 b | 0.028 b | 0.21 b | 0.56 b |

Numbers followed by different letters are significantly different (p < 0.05).

**Supplementary** **Table S4**. Analysis of variance for effects of RERP on peach fruit size, weight and quality

| Treatment | Transverse Diameter (mm) | Vertical Diameter (mm) | Single Fruit Weight (g) | Nitrate Content (mg kg^-1^) | Vitamin C (mg g-1) | Soluble Sugar (%) | Titratable Acid (%) | Total Soluble Solid (%) |
| --- | --- | --- | --- | --- | --- | --- | --- | --- |
| CK | 70.10 b | 65.79 b | 168.50 b | 119.79 a | 58.84 b | 8.48 b | 2.35 a | 9.64 b |
| T1 | 73.99 a | 71.10 a | 196.04 a | 90.40 c | 93.00 a | 10.26 a | 1.51 b | 10.58 a |
| T2 | 73.56 a | 73.33 a | 179.12 b | 103.14 b | 64.53 b | 9.50 a | 2.01 a | 10.55 a |

Numbers followed by different letters are significantly different (p < 0.05).

**Supplementary** **Table S5**. Analysis of variance for alpha diversity estimates of soil bacterial community in root zone of peach trees

| Treatment |  | Diversity | |  | Richness | |
| --- | --- | --- | --- | --- | --- | --- |
|  | Sobs | Shannon | Simpson |  | Chao1 | ACE |
| Cka | 3698 c | 6.52 b | 0.0067 a |  | 4982 c | 4922 c |
| CKb | 3375 d | 6.46 b | 0.0038 c |  | 4655 c | 4953 c |
| T1a | 4423 a | 6.82 a | 0.0043 bc |  | 6045 a | 6058 a |
| T1b | 4385 a | 6.82 a | 0.0042 bc |  | 5956 a | 5935 ab |
| T2a | 4044 b | 6.71 a | 0.0049 bc |  | 5477 b | 5459 bc |
| T2b | 3263 d | 6.50 b | 0.0053 ab |  | 4620 c | 5106 c |

Numbers followed by different letters are significantly different (p < 0.05).
